# In memory of Professor Thomas Wieland (1960–2025)

**DOI:** 10.1007/s00210-025-04735-2

**Published:** 2025-10-27

**Authors:** Bernd Nürnberg, Issam H. Abu-Taha, Susanne Lutz, Martina Schmidt

**Affiliations:** 1 Research Training Group 2816, Department of Pharmacology, Experimental Therapy & Toxicology, and Interfaculty Center of Pharmacogenomics & Drug Research (IZePhA), University Hospitals and Clinics, Eberhard-Karls University Tübingen, Tübingen, Germany; 2 Institute of Pharmacology, West German Heart and Vascular Center, Faculty of Medicine, University Duisburg-Essen, Essen, Germany; 3 Institute of Pharmacology and Toxicology, University Medical Center Göttingen, Göttingen, Germany; 4 Department of Molecular Pharmacology, Groningen Research Institute of Pharmacy (GRIP), University of Groningen, Groningen, The Netherlands

**Keywords:** Exchange factor, GPCR, G protein, NDPK, NME


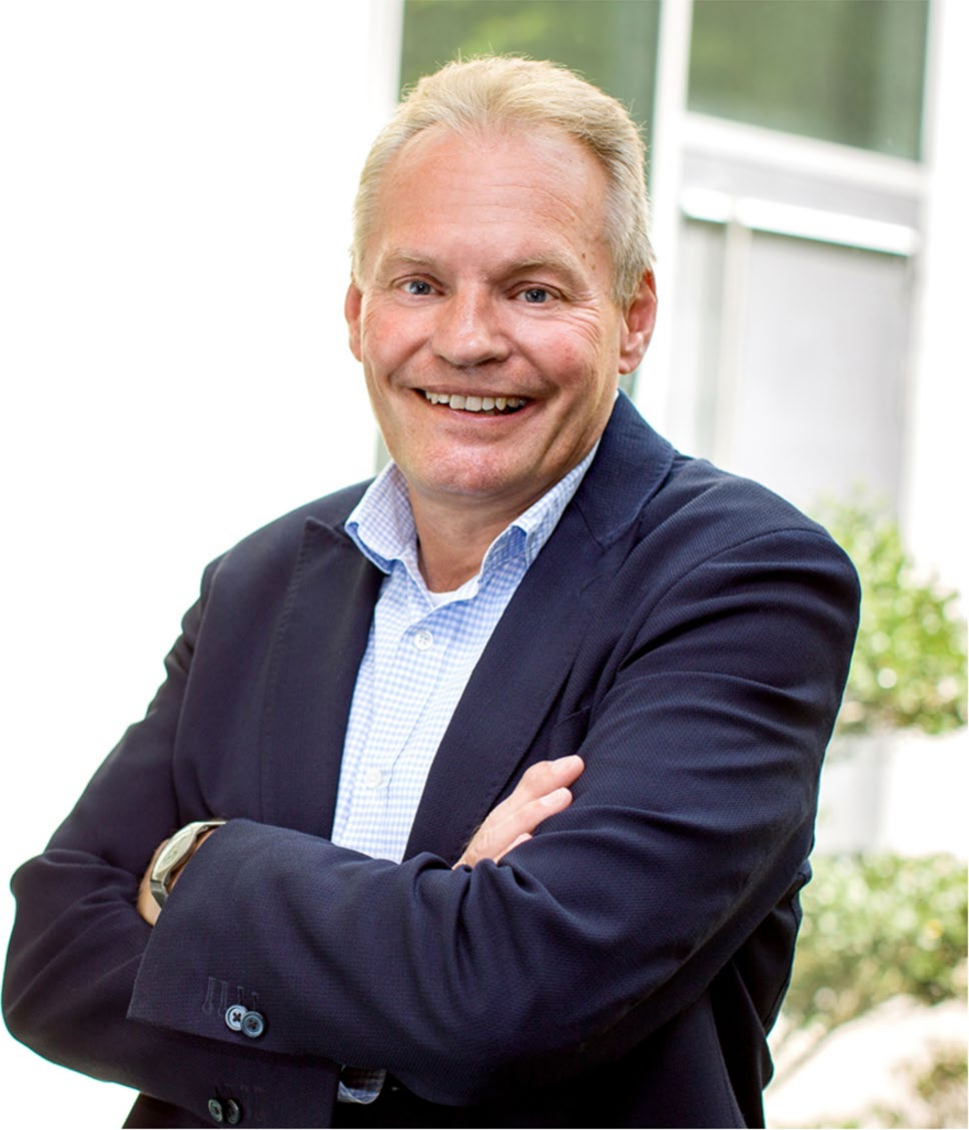


When we came together in Mannheim on April 9 of this year to honor Professor Dr. Thomas Wieland with the *Schmiedeberg Medal* of the German Society for Experimental and Clinical Pharmacology and Toxicology (Lutz et al. 2025), we encountered a laureate who, though visibly affected by illness, faced it with extraordinary courage and dignity. He exuded confidence, spoke with conviction about future plans, and confronted a relentless disease with determination and hope. Only a few months later, we are left with the painful task of bidding farewell to a leading figure in German and international pharmacology, whose scientific achievements, personal integrity, and inspiring mentorship will remain an enduring legacy for generations to come.

Thomas Wieland was born on September 14, 1960, in Karlsruhe as the first son of Renate Wieland who, as was common in West Germany at the time, devoted herself to the family as a housewife, and Horst Wieland who managed his own business as an independent merchant. He grew up in nearby Ettlingen together with his younger brother, who sadly passed away in 2000, and a sister born in 1970. There he spent his school years and graduated from high school (Abitur) in 1980. As was customary at the time, he then completed 15 months of compulsory military service in the 1st Airborne, serving as a member of the paratrooper corps. This experience reflected his discipline, resilience, and commitment to teamwork.

Already in his school years, Thomas Wieland was deeply fascinated by chemistry, a passion that led him to begin studying pharmacy at Heidelberg’s *Ruperto Carola* in the winter semester of 1981/1982, despite his father’s having envisioned a future for him in the family business. What captivated Thomas most about pharmacy was the central role of chemistry—woven into the broader fabric of the natural and life sciences—which opened up a wide horizon of academic and scientific discovery. Even before entering university, his curiosity about how drugs work was so strong that he purchased a pharmacology textbook and read it with great enthusiasm. He was also fortunate to be guided by Pharmacology Professor Eberhard Hackenthal (1931–2013), an inspiring teacher who fostered Thomas’s passion for the field with remarkable ease and elegance (Wieland 2017). In 1985, he successfully completed his studies with the second state examination in Pharmacy and went on to undertake his practical training year at a public pharmacy in Mannheim. Having passed the final state examination and obtained his license as a pharmacist, he mounted his motorcycle and set off for Florence to spend 3 months with his future wife, Claudia, who was then studying medicine there—a journey that reflected both his zest for life and, after times of separation, his deep longing for her. It was in Florence that their shared love for Italy first began to take root.

Back in Germany, Thomas Wieland knocked on the door of Professor Karl-Heinz Jakobs (1941–2018) at the Pharmacological Institute of Heidelberg University searching for a doctoral position—and Jakobs welcomed him without hesitation. This step in the fall of 1986 marked the beginning of an exceptionally fruitful collaboration between two scientists who were remarkably attuned to one another, a partnership that soon took on the character of an academic father–son relationship.

Jakobs himself had been shaped by the scientific school of Professor Günter Schultz (1936–2021), the pioneer of G protein research in Germany. When Schultz was appointed Chair of Pharmacology at the Free University of Berlin, Jakobs succeeded him as C3 Professor in Heidelberg (Aktories et al. 2019). His passion lay in the elegant and precise dissection of G protein-regulated mechanisms of adenylyl cyclase. Among his pioneering insights was the concept of an inhibitory G_i_ protein acting on adenylyl cyclase. He was equally fascinated by the idea that a membrane-associated form of nucleoside diphosphate kinase (NDPK) could form a complex with heterotrimeric G proteins and, through this alternative activation pathway, supply GTP to GTPases. Jakobs was the first to extend this concept to G_i_ proteins and their inhibitory regulation of adenylyl cyclase.

As a young doctoral student supported by a graduate scholarship from the state of Baden-Württemberg, Thomas Wieland followed his supervisor and developed a profound fascination with the function, regulation, and biological significance of NDPKs—a passion that would accompany and inspire him throughout his entire scientific career. Thomas Wieland later reflected on this enduring interest in his obituary for his doctoral mentor, Karl-Heinz Jakobs (Aktories et al. 2019), with further details also highlighted in the recent laudatory address dedicated to Thomas (Lutz et al. 2025). His very first scientific presentation, delivered at one of the legendary spring meetings of the German Pharmacological Society (DGPT) in the distinctive “auditorium shell” (*Hörsaalmuschel*) of Mainz University in 1988, was devoted to this subject (T. Wieland: *Receptor-stimulated formation of GTP by nucleoside diphosphokinase (NDPK) in membrane preparations*; Abstract 155).

That same year, he appeared as co-author of a joint publication with the Schultz group in Berlin—an early contribution that, through decades of collaboration and shared publications, earned him the affectionate recognition as an “academic grandson” of Günter Schultz (Seifert et al. 1988). Two further articles, published in 1989 in sole authorship with his doctoral advisor, paved the way for the completion of his dissertation entitled “Activation of signal-transducing guanine nucleotide-binding proteins: Investigations into the involvement of transphosphorylation reactions” (original German title: “*Aktivierung signalübertragender, Guaninnukleotid-bindender Proteine: Untersuchungen zur Beteiligung von Transphosphorylierungsreaktionen*”), with which he was awarded the degree of *Doctor rerum naturalium* with *summa cum laude* honors at the end of 1989.

With the completion of his doctorate and a first postdoctoral position in Jakobs’ group, Thomas Wieland had reached a stage of professional accomplishment that, maintaining the family traditions of the time, allowed him to marry his beloved Claudia. Scientifically, he now began to broaden his horizons, turning his attention to the so-called “small” monomeric GTPases. His findings soon appeared in print, and within a short time, his contributions were being published with notable frequency, particularly in the *European Journal of Biochemistry.*

In 1991, Thomas Wieland’s mentor, Karl-Heinz Jakobs, accepted the chair of pharmacology at the Medical Faculty of Essen, and Thomas followed him as a young group leader. His first publication with an Essen affiliation, addressing the binding affinities of GDP to G proteins, appeared in May 1992 and was soon followed by numerous other papers that allowed him to mature into an independent scientist recognized within the international signal transduction and G protein community. His standing became particularly visible within the DFG (Deutsche Forschungsgemeinschaft) Infrastructure Priority Program (SPP) on Molecular Mechanisms of Signal Transduction in Membranes (“*Molekulare Mechanismen der Signaltransduktion in Membranen*”) coordinated by Günter Schultz and running from 1988 to 1994 with annual colloquia in Berlin each February. Whereas Jakobs initially presented Wieland’s results in 1991, Thomas himself took the stage at the 4th and 5th colloquia in 1993 and 1994, reporting on the role of Gβ subunits in phosphate transfer and the involvement of microfilaments in receptor–G protein coupling. Even after the Berlin colloquia gave way to the Dahlem colloquia of Collaborative Research Center (SFB) 366 Cellular Signal Recognition and Translation (“*Zelluläre Signalerkennung und -Umsetzung*”), he remained a welcome speaker, presenting in October 1995 on phosphorylation reactions. His trajectory culminated in November 1995 with his habilitation in pharmacology and toxicology at the Faculty of Medicine in Essen, based on his thesis on Molecular Mechanisms of Activation of Heterotrimeric Guanine Nucleotide-Binding Proteins (“*Molekulare Mechanismen der Aktivierung heterotrimer Guaninnukleotid-bindender Proteine*”)*.*

During these years, his private life was no less eventful. The family grew with the birth of their first daughter Anne in 1990, and Claudia pursued her own medical career in nearby Duisburg, first as a PJ (praktisches Jahr) student, later as an AiP (Arzt im Praktikum), and then as a resident physician, allowing Thomas to devote himself fully to his scientific work. Soon after, supported by a DFG fellowship, Thomas decided to broaden his scientific spectrum abroad. With his young family at his side, on April 1, 1996, he joined the laboratory of Professor Melvin I. Simon at Caltech in Pasadena, California—then a hot spot for G protein research—to study the biological roles of G proteins by generating gene-deficient mouse lines. During this time, he also met Dr. Ching-Kang Jason Chen (now Professor at San Antonio, TX, USA) and collaborated with him on pioneering studies of the newly identified regulator of G protein signaling (RGS), RGS16. Although his stay lasted less than originally anticipated, it proved highly productive, yielding two publications in the *Journal of Biological Chemistry* and *Proceedings of the National Academy of Sciences USA*. In Pasadena, the family was blessed with the birth of their second daughter Johanna.

When he was offered a C3 professorship at the Institute for Experimental and Clinical Pharmacology and Toxicology at the University Medical Center Hamburg-Eppendorf, led by Professor Hasso Scholz and focused on cardiovascular pharmacology, the opportunity was so compelling—yet limited in time—that in the fall of 1997 he left Caltech earlier than intended to accept the position.

Thomas Wieland’s years in Hamburg were marked by a decisive shift in his research that both catalyzed his scientific development and paved the way for his later success. Whereas his earlier work had focused primarily on the fundamental mechanisms of G protein-dependent cellular signaling, he now began to integrate these foundations with innovative, pharmacologically oriented cardiovascular research. On the occasion of receiving the *Schmiedeberg Medal* on April 9, 2025, he expressed his gratitude to Professor Thomas Eschenhagen (T.E.), Hasso Scholz’s former student and successor to the Hamburg chair of pharmacology, in moving words: “You (T.E.) opened the door for me and showed me what was important. I came from biochemistry, I came from the basics, and then I tried to understand what you had done” (“*Du (T.E.) hast das Tor für mich geöffnet und mir demonstriert, was wichtig war. Ich kam aus der Biochemie, ich kam aus den Grundlagen und habe dann versucht zur verstehen, was Ihr gemacht habt*”)*.*

While cellular signal transduction remained the scientific foundation of his work, he expanded his focus to include the pharmacological implications of these mechanisms. In 1998 and 1999, he presented his findings on regulators of G protein signaling (RGS proteins) at the 3rd and 4th colloquia of the DFG priority program on GTPases as Central Regulators of Cellular Functions (“*GTPasen als zentrale Regulatoren zellulärer Funktionen*”), coordinated by Professor Peter Gierschik, Ulm, at Reisensburg Castle in Günzburg. At the same time, he increasingly published on the role of GPCR (G protein-coupled receptor)-, G protein-, and RGS-regulated mechanisms in cardiomyocytes under both physiological and pathophysiological conditions.

In Hamburg, he proved himself not only as a favored and valued collaborator for several cardiology-focused pharmacologists and a dedicated doctoral supervisor, but also an inspiring mentor, with Friederike Cuello—later the first of several of his students to attain a professorship—standing as a testament to his guidance. His excellent reputation was soon acknowledged with an offer of a tenured C3 professorship in pharmacology and toxicology in Essen in 2001, which he declined in order to accept a simultaneous offer from Mannheim.

Yet, these years of professional success came at a personal cost. The Wieland family had meanwhile settled in their hometown of Ettlingen, and for 5 years, Thomas commuted between his home near Karlsruhe and Hamburg—a demanding rhythm that reflected both his devotion to his family and his unrelenting commitment to science.

A new chapter began in summer 2002, when Thomas Wieland was appointed to an independent, tenured W3 professorship at the Institute of Pharmacology and Toxicology of the Medical Faculty in Mannheim—his old *alma mater*, Heidelberg’s *Ruperto Carola*. This move marked not only a major professional milestone but also a personal relief, as Mannheim brought him much closer to the family’s home in Ettlingen after years of long commutes.

In the years that followed, he received further calls to chairs of pharmacology in pharmacy, including offers from the University of Braunschweig in 2004 and the University of Frankfurt in 2014, both of which he declined. Although by then firmly rooted in medicine, he continued to feel a strong bond with his original discipline of pharmacy. A telling example was his contribution to the joint annual meeting of the German Pharmaceutical Society (DPhG) together with the Czech and Hungarian Pharmaceutical Societies in Marburg in 2006, where he talked about what had long since become “his” subject—the NDPK.

His colleagues in Mannheim quickly came to value both his scientific stature and his personal integrity, and they were determined not to let him go. In 2007, Thomas Wieland succeeded Professor Björn Lemmer and was appointed Chair and Director of the Institute for Experimental and Clinical Pharmacology and Toxicology, a position in which he would shape the field for the years to come. In Mannheim, Thomas Wieland built on his broad scientific foundation to establish a lasting focus on translational cardiovascular pharmacology, which he pursued with determination and intellectual clarity. He also earned recognition as both a true artist of G-protein research and a bridge builder between basic science and clinical translation. At the center of these efforts stood the integration of his basic research on guanine nucleotide–regulated signal transduction.

Very early at that time, Susanne Lutz joined him as a postdoctoral fellow from Professor Feraydoon Niroomand, cardiology department in Heidelberg, and together they pushed forward his favorite NDPK research in close collaboration with Niroomand and later with his student, the later Priv.-Doz. Hans-Jörg Hippe. With persistence and through fruitful collaborations with renowned partners, he demonstrated the relevance of NDPKs—long regarded merely as “housekeeping” enzymes—for (patho)physiological signaling processes (Lutz et al. 2025). While Professor Alfred G. Gilman (1941–2015), the Nobel laureate and icon of G protein research, still spoke somewhat mockingly in the 1990s to Günter Schultz about NDPKs as cellular signal mediators, remarking, “I can live without them,” Thomas Wieland remained undeterred. He went on to decipher crucial regulatory functions of these multifunctional histidine NDPKs in the heart, particularly in heart failure—together with Professor Dobromir Dobrev (now Essen)—, in the vascular endothelium, and in oncology with regard to tumor growth and metastasis. Convinced of their potential as novel pharmacological targets in cardiovascular and oncological diseases, he successfully worked with Professor Klaus Scheffzek’s group in Innsbruck to resolve the three-dimensional structures of NDPKs as a precondition for design-based drug development. Moreover, he identified NDPK inhibitors as potential lead compounds in drug screening approaches. His work has substantially deepened our understanding of NDPKs in the regulation of vital functions in both health and disease.

At the same time, he remained committed not only to expanding his “G-research” but also to embracing new methods and techniques. Thomas Wieland began studying Rho-type monomeric GTPases early in his career—key regulators of the cytoskeleton—and over the course of his scientific life published more than 30 papers on this subject. As noted above, he had also initiated studies on RGS proteins as a novel class of G protein regulators during his time in Pasadena. These two strands of research converged in Hamburg, where he demonstrated G protein-mediated activation of Rho GTPases via guanine nucleotide exchange factors (GEFs). In this context, Thomas Wieland was able to show that GEFs such as p63RhoGEF, Kalirin, or Trio mediate RhoA activation through direct interaction with activated Gα_q/11_ (Wieland 2017), and he published the finding that this mechanism underlies angiotensin II–dependent contractility and proliferation of vascular smooth muscle cells. In a highly fruitful collaboration with Professor John Tesmer (West Lafayette, IN, USA) and together with Susanne Lutz, he later elucidated the structure of the Rho-activated Gα_q_–p63RhoGEF–RhoA complex, a milestone published in *Science* in 2007 (Lutz et al. 2007). Beyond these landmark achievements, he continued to investigate a wide range of physiologically relevant interactions between heterotrimeric and monomeric GTPases and their regulators. Thomas Wieland became a central figure within the international NME/NDPK/NM23/AWD gene family community and assumed a leading role in organizing its triennial international conferences, including the landmark 2010 meeting in Mannheim. Tangible recognition of his outstanding scientific achievements came through academic distinctions such as the Albert Fraenkel Award in 2003 and the Honorary Award Lecture on Basic Science of the German Society of Cardiology in 2016.

Thomas Wieland was a passionate team player who not only cultivated many successful individual collaborations—often supported by joint DFG-funding—but also contributed continuously for decades to DFG-funded research consortia such as SPPs, Research Training Groups (RTG), and CRCs. In addition, the BMBF (Federal Ministry of Education and Research) and especially the DZHK (German Centre for Cardiovascular Research) were vital sources of support for his projects. The DFG also provided him with resources to organize scientific conferences, while BMBF funding enabled him to advance teaching innovations, such as the development of an intranet-based learning system on coronary heart disease.

Thomas Wieland was deeply engaged in the scientific community, where he combined leadership, vision, and a strong sense of responsibility. In Mannheim, he shaped the cardiovascular focus and integrated his institute into the European Center for Angioscience, while also serving as Deputy Partner Site Speaker for the DZHK. Within the DFG, he contributed for many years as a member and also as Deputy Speaker of the Medical Review Board, and in pharmacology, he left his mark as long-standing editor of *Naunyn–Schmiedeberg’s Archives of Pharmacology* and for years as Vice President of the German Society for Pharmacology (DGP), before becoming its President in 2018. He tirelessly promoted international exchange, culminating in the successful bid to bring the EPHAR Congress 2027 to Germany—an achievement he sadly will not live to witness. With the same dedication, he embraced academic self-governance, most notably as Dean of Studies in Mannheim from 2016 onward, a role that reflected his deep passion for teaching and his joy in supporting the next generation of scientists.

All these efforts demanded an exceptional degree of personal dedication and came with a workload that drew deeply on his energy. At the same time, he and his wife had cultivated a lasting affection for Italy since Claudia’s student days. This found expression in a family retreat on Lago Maggiore—a place of rest and recreation where he enjoyed water sports such as boating and diving and a cherished refuge each summer. There he could withdraw in privacy, reflect, and work creatively on ideas, grant proposals, or manuscripts, free from distraction.

Thomas Wieland combined in a rare way the qualities of a passionate, brilliant researcher and academic teacher with those of a devoted husband, father, and grandfather, for whom family always came first. As we mourn the loss of a distinguished scientist who made lasting contributions to German pharmacology, our heartfelt condolences go out to his family. His life and work will remain an enduring inspiration to pharmacology in Germany and beyond.

## Personal perspective of a close colleague from his time in Essen (MS)

My personal journey with Thomas started on April 1, 1993. Thomas had started as a junior group leader at Professor Karl-Heinz Jakobs’ Institute in Essen in 1991. During this period, Thomas Wieland explained with seemingly never-ending energy and patience the “secrets” of GPCR signaling to us, a group of early career scientists. I was one of the unexperienced post-docs. Thomas succeeded in explaining the cycling of G proteins between an inactive and an active state and in transferring the knowledge of how to measure these different activity stages. Our first publication together established a novel method to study receptor–G protein interactions and has been published in *Naunyn–Schmiedeberg’s Arch. Pharmacol* in 1995 (Wieland et al. 1995). In the same year, Thomas was instrumental for our publication showing that receptor signaling to phospholipase C and phospholipase D followed a very distinct pattern (Schmidt et al. 1995).

Thomas went for a research stay at the prestigious California Institute of Technology in 1996, and he broadened his research focus and started to work on RGS family of proteins. Thanks to his new research focus, we characterized the pertussis toxin-insensitive coupling of a differential subset of effector proteins to prototypical GPCRs (Rümenapp et al. 2001; Lutz et al. 2005; Vogt et al. 2007). During this period, Thomas succeeded in bridging any differences in scientific views and personalities, without which these publications would not have been possible. The research focus shifted a bit away from the RGS proteins to GEFs for the monomeric GTPases Rho and Rac. Our very productive research interaction also included discussions about yet neglected positive interactions between receptors elevating calcium and those elevating cyclic AMP. A key protein here was the cAMP-regulated exchange factor EPAC. Thomas not only pushed his research ideas about the function of the NDPK-B isoform and its role in the regulation of endothelial cell integrity ahead (Chatterjee et al. 2020a,b), but he also supported new insights into the role of EPAC in phenotypical alterations of both epithelial and endothelial cells (Jansen et al. 2016; Garg et al. 2017). Several of my doctoral students and junior postdocs, including Sepp Jansen and Jaspal Garg, greatly benefited from Thomas’ creative perspective on science, and I was deeply grateful for his continuous support.

During all these years we kept contact, sometimes we had seen each other more frequently, and sometimes we only had phone calls. But our common research interest continued, and due to the friendship, which had developed over the years, we never stopped with our endless fruitful discussions. We met each other at several national and international conferences. We were both part of the International training network which was established between Mannheim and Groningen (DIAMICOM). We exchanged students, and we reached out to each other when needed.

I deeply regret that Thomas Wieland, who never gave up even if situations were challenging, lost his personal fight against cancer. He passed away much too early. We will try to do our best to fulfill in the future the high standard and personal touch Thomas Wieland had given to everything. I have lost a friend!

## Personal perspective of a mentee (SL)

My mentor and friend, Thomas, has influenced me since long before we met in person, and he will continue to do so for the rest of my life.

It all began when I was a young doctoral student studying the role of NDPK in heart failure in Professor Feraydoon Niroomand’s group in the Department of Cardiology at Heidelberg University Hospital (1997–2002). In addition to working countless hours in the cold room and staring hypnotically at the liquid scintillation counter, I spent a lot of time in the dusty university library photocopying relevant literature. It was there that I came across the work of “Wieland, T.” This was not surprising, given that “Wieland, T” had authored the most relevant publications for my thesis and had demonstrated over 10 years earlier that NDPK could induce receptor-independent activation of heterotrimeric G proteins. Similarly, we found that NDPK inhibited adenylyl cyclase via G_i_ proteins in the failing heart.

Despite my thesis work being closely related to that of “Wieland, T,” I never met him in person during those years. However, towards the end of my thesis, Feraydoon mentioned that Thomas Wieland was going to take up a W3 position in pharmacology in Mannheim and was looking for a postdoc. Feraydoon gave me Thomas’s number, and I nervously called him on a Saturday. We arranged to meet in person. It was April, a sunny day; the annual meeting of the German Society for Cardiology was taking place. We met on a bench in front of the Rosengarten. He smiled at me and the highly respected scientist I knew as “Wieland, T” became my boss, my mentor, and my friend Thomas Wieland.

On this bench, Thomas offered me a postdoc project on guanine nucleotide exchange factors. After both of us agreed that I would become his postdoc in 2002, we began setting up our laboratory in Mannheim. It was located above a children’s clothing store, next to a supermarket and the Mannheim fairground. It was not the place where you would ever expect to find an institute of pharmacology. At first, all we had was space, but over time, we organized and established most of the things we needed, and Thomas was excellent at organizing things out of nowhere.

One of our greatest treasures was our ability to communicate. Talking with Thomas about scientific projects was like participating in a table tennis championship. You had to know your facts before stepping into the tournament. I never experienced that again with anyone else. However, this is, in my opinion, the best way to motivate young scientists to dive deeper into a topic, especially when there is the chance to convince your experienced “opponent” of your ideas.

So, we were not one of those wealthy working groups with the latest machines in a vibrant setting. However, we were passionate about what we did. Perhaps this is why we were able to convince highly motivated medical and doctoral students to join us in our enclave. The rooftop barbecues probably helped, too. It was really fun!

Time flew past. We worked hard, with pleasure and some success, and like many postdocs in this situation, I couldn’t imagine that things might have to change. Then, Thomas, now showing his mentor qualities, started asking me to give a few lectures. He said that the toxicology of pesticides might be appropriate for a trained biologist—believe me, it is not—but he probably thought that medical students would not be interested in challenging a nervous lecturer, who was clearly not medically trained, on this topic. That’s how my career as a lecturer began, and I still love doing it.

In 2006, Thomas suggested that I spend a few weeks at John J. G. Tesmer’s laboratory in Ann Arbor, Michigan. He thought it would be exciting to crystallize p63RhoGEF with Gα_q_, but well, being a cell biologist, I was not immediately convinced. Nevertheless, the next day, I agreed. In retrospect, I believe this experience was of the utmost importance for both of us. In a short time, we collected together with John’s group enough data to publish, and Thomas negotiated the author list with John. The paper was published in *Science* in 2007 (Lutz et al. 2007). Almost 20 years later, with a better understanding of the realities of research, it is very likely that these negotiations were not easy and it is very clear that Thomas acted in my favor.

It’s not surprising that such a paper opens doors. Thomas made sure the door opened for both of us, even if it meant having two separate doors. This is often when the real problem starts: What’s mine and what’s yours? We never had these problems because Thomas was extremely generous. He said, “Take the p63RhoGEF story, take the mouse…,” so I did. When I moved to Göttingen in 2010 to establish the “GPCR Signaling Research Unit” and, a year later, to assume a professorship in biochemical pharmacology, I did so with mixed emotions—leaving behind the familiar was not easy, but I was eager to embrace the new opportunities and challenges that lay ahead. The disadvantage of not “divorcing” with a War of the Roses is that the mentee’s independence from the mentor is sometimes questioned. Well, nothing comes for free.

Young students often ask me what is necessary for an academic career. My answer is always the same: “You must love what you do. You also need some luck, and the best luck you can have is to have a mentor like mine.” Today, I know that Thomas wove me from early on into his silky net without my knowledge, and at the right moment, he pulled the right string to open the right door.

At least, I try to follow in his footsteps.

## Personal perspective of a former doctoral student (IA-T)

Thomas Wieland leaves behind an extraordinary scientific and personal legacy. His pioneering work transformed the field of pharmacology, and his mentorship shaped the lives of countless colleagues and students. He was an outstanding mentor and supervisor who consistently encouraged his students to be independent, to develop their own ideas, and to contribute creatively to collaborative projects. He generously supported interdisciplinary work and career development. Thomas treated his students and colleagues with respect, empathy, and fairness, fostering an atmosphere of openness and intellectual exchange. For him, mentorship meant empowerment and guidance without imposing hierarchy, always combined with attentive listening. Above all, Thomas was a humanist. He believed in equality of opportunity, regardless of origin or background, and offered his support without prejudice or reservation.

As a doctoral student, I had the privilege of working closely with Thomas between 2007 and 2011. Our story began when I knocked on his office door searching for a new doctoral position. Without hesitation, he welcomed me into his lab. A few months later, he handed me a special coin—a Pfennig of the Bank of German States—and told me it should be my lucky charm. It is a gesture that I am still thinking about to this day, and I still keep that coin with me.

After about a year, he encouraged me to start working more independently and reminded me that my own ideas should shape the NDPK project I was working on. I vividly remember the first time I presented my results on NDPK-C. He was excited and visibly moved, and later Professor Susanne Lutz confirmed his reaction by saying: “Thomas, Issam has just proven what you were seeking.” It was a moment that revealed his passion for science—a scientist constantly searching for answers, never losing his curiosity.

Thomas was also active and eager to share knowledge directly at the bench. I once asked him to join me in the lab, and together, we performed radioactive experiments showing how NDPKs interact and contribute to the activation of the retinal G protein transducin (G_tαβγ_) he had previously isolated from bovine retina during his time in Essen. That was a moment of high-quality time; I learned not only technical methods but also his way of approaching science with rigor and joy. For Thomas, the lab was like a family. He gave his students the feeling of belonging, of being part of something larger. Even outside the lab—whether during barbecues on the institute’s roof, where he happily prepared food for everyone, in beer gardens, or in daily interactions—he treated us with warmth and camaraderie.

The years between 2007 and 2011 were truly formative, both scientifically and personally. They were filled with countless discussions, often about science, but also about life more broadly. These years were very fruitful, and together, we uncovered how NDPK-B anchors and regulates G proteins at the plasma membrane, work that led to multiple publications (Hippe et al. 2009; Hippe et al. 2011a,b). His mentorship during those years, and long afterwards, profoundly shaped my scientific career.

Under his and Professor Dobromir Dobrev’s supervision—to whom Thomas recommended me, and with whom I have continued to work ever since—we identified critical roles of NDPK-C in G protein signaling and cardiac function. We demonstrated that NDPK-C is the limiting factor for the interaction of NDPK-B with G proteins and that NDPK-C switches from Gα_s_ to Gα_i2_, thereby reducing cAMP levels in heart failure. These insights culminated in our *Circulation* publication in 2017 (Abu-Taha et al. 2017). This was only possible because Thomas gave me the freedom to experiment and to pursue new ideas, even when he himself was not fully convinced they would succeed.

Over the years, we stayed in close contact. He treated me as a friend, and we regularly discussed science as well as life beyond it. He supported me in all my endeavors. We had planned to attend the next NDPK conference in October 2025 in Budapest together; now, sadly, I will go alone and give a talk in his honor. His final words to me were filled with encouragement to pursue my habilitation and to carry on with the NDPK project.

We have lost not only a brilliant scientist but also an extraordinary mentor and a dear friend. What Thomas Wieland gave to science will endure. What he gave to people—respect, inspiration, and kindness—will remain with us. His legacy will live on in our research and in our thoughts.

## Personal perspective of a colleague and friend (BN)

For 33 years, Thomas Wieland was to me not only a close and trusted colleague, advisor, and collaborator, but above all a dear friend. I vividly remember the beginning in 1992, when Karl-Heinz Jakobs called Günter Schultz to ask him about the possibility of demonstrating the phosphorylation of G-protein β subunits in cell membranes. Schultz agreed and invited me to collaborate—an extraordinary stroke of fortune for me, both scientifically and personally, as it marked the start of a partnership and friendship that profoundly shaped my career.

After 7 years of work in pharmacokinetics and applied pharmacological research, I had only recently changed my scientific direction rather radically and was urgently seeking exchange and collaboration with competent partners in this new field from whom I could learn as much as possible. Thomas Wieland was the first from whom I benefitted enormously—even though we were geographically apart, with him in Essen and me in Berlin. And we quickly found success: in November 1992, a manuscript was submitted to the *Journal of Biological Chemistry* (*JBC*), and by the time of Thomas’s already mentioned lecture at the 4th Berlin SPP Colloquium on February 25, 1993, I was listed as a co-author. The paper was under revision at that time and was published in the *JBC* on August 25, 1993 (Wieland et al. 1993). I was both surprised and especially pleased to be named as second author. And not only that: another gesture of support came in the form of an invitation to give a talk in Essen on February 15, 1994—where it went without saying for Thomas that I should stay overnight at his home. Our collaboration and our scientific exchange continued, and 2 years later, we reported together on the Gβ phosphorylation in different tissues. At that point, Thomas was already habilitated and preparing for his move to Mel Simon’s lab at Caltech, while I was pursuing my own habilitation—so I was very glad for our joint paper, with him as last author and me as first. As a side note, the placenta used in those studies had been “donated” by my wife and our daughter Christina.

Even though the number of our joint publications was limited, we soon discovered how wide the spectrum of our shared views and values truly was. We exchanged ideas, sought each other’s advice, and offered support whenever possible. I admired him for his passion for tinkering, in both his research and his private life, where perseverance was simply part of who he was. During the period when both of us were being considered for professorships, we might have been seen as competitors, but I never regarded him that way. I particularly remember a call when he asked for my advice about accepting the position of Dean of Studies, trusting my experience as Vice Dean and Dean in Düsseldorf. Conversely, he readily supported and joined my RTG initiative, fully aware that his benefit would be small—yet he agreed without hesitation. I will always remember Thomas’ last appearance at our RTG symposium at Lake Ammersee in September 2024, when he seemed joyful and carefree, even as the first symptoms were already casting their shadow and Claudia had begun arranging medical appointments for him (Haas et al. 2025).

I came to know Thomas Wieland as an outstanding scientist who was a sharp-minded, steadfast, and ambitious distinguished figure in the best sense. He was calm and objective, never irritable, and marked by rare modesty, thoughtfulness, and self-restraint. His career shows him as a bridge builder in Science, breaking down barriers between groups and dogmata, working selflessly for the sake of science. I am profoundly grateful to have had such an exceptional person, both scientifically and personally, as a friend in my life.

## Data Availability

No datasets were generated or analysed during the current study.
